# Genome‐wide aneuploidy detected by mFast‐SeqS in circulating cell‐free DNA is associated with poor response to pembrolizumab in patients with advanced urothelial cancer

**DOI:** 10.1002/1878-0261.13196

**Published:** 2022-03-17

**Authors:** Pauline A. J. Mendelaar, Debbie G. J. Robbrecht, Maud Rijnders, Ronald de Wit, Vanja de Weerd, Teoman Deger, Hans M. Westgeest, Maureen J. B. Aarts, Jens Voortman, John W. M. Martens, Astrid A. M. van der Veldt, José Alberto Nakauma‐González, Saskia M. Wilting, Martijn Lolkema

**Affiliations:** ^1^ Department of Medical Oncology Erasmus MC Cancer Institute Rotterdam The Netherlands; ^2^ Department of Medical Oncology Amphia Hospital Breda The Netherlands; ^3^ Department of Medical Oncology GROW School for Oncology and Developmental Biology Maastricht University Medical Center+ The Netherlands; ^4^ Department of Medical Oncology Cancer Center Amsterdam Amsterdam UMC, Vrije Universiteit Amsterdam The Netherlands; ^5^ Department of Radiology & Nuclear Medicine Erasmus MC Cancer Institute Rotterdam The Netherlands; ^6^ Department of Cancer Computational Biology Center Erasmus MC Cancer Institute Rotterdam The Netherlands; ^7^ Department of Urology Erasmus MC Cancer Institute Rotterdam The Netherlands

**Keywords:** advanced urothelial carcinoma, biomarker, chromosomal instability, ctDNA, liquid biopsies, pembrolizumab

## Abstract

Second‐line treatment with immune checkpoint inhibition in patients with metastatic urothelial cancer (mUC) has a low success rate (~ 20%). Circulating tumour‐derived DNA (ctDNA) levels may guide patient stratification, provided that an affordable and robust assay is available. Here, we investigate whether the modified fast aneuploidy screening test‐sequencing system (mFast‐SeqS) may provide such an assay. To this end, mFast‐SeqS was performed on cell‐free DNA (cfDNA) from 74 patients with mUC prior to treatment with pembrolizumab. Results were associated with corresponding tissue‐based profiles, plasma‐based variant allele frequencies (VAFs) and clinical response. We found that plasma‐derived mFast‐SeqS‐based aneuploidy scores significantly correlated with those observed in the corresponding tumour tissue as well as with the ctDNA level in the plasma. In multivariate logistic regression analysis, a high aneuploidy score was independently associated with lack of clinical benefit from treatment with pembrolizumab. In conclusion, mFast‐SeqS provides a patient‐friendly, high‐throughput and affordable method to estimate ctDNA level. Following independent validation, this test could be used to stratify mUC patients for response prior to the initiation of treatment with pembrolizumab.

## Graphical abstract

Second‐line immune checkpoint inhibition in patients with metastatic urothelial cancer (mUC) has a low success rate. ctDNA levels may guide patient stratification; however, a high‐throughput and affordable method is lacking. Here, we report that a high aneuploidy score detected by the mFast‐SeqS method is independently associated to a lack of clinical benefit from pembrolizumab treatment. Following independent validation, mFast‐SeqS could stratify patients with mUC for response to therapy.

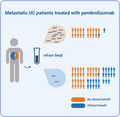

**Abbreviations**
cfDNA: cell free DNA
CNA(s): copy number alteration(s)
CPS: combined positivity score
ctDNA: circulating tumour DNA
FFPE: formalin‐fixed and paraffin‐embedded
ICI: immune checkpoint inhibitor
mFast‐SeqS: modified Fast Aneuploidy Screening Test‐Sequencing System
mUC: metastatic urothelial cancer
NGS: next generation sequencing
VAF(s): variant allele frequency (frequencies)
WGS: whole genome sequencing

## 1 Introduction

Stratifying which patients might benefit from systemic treatment prior to the start of a certain therapy is necessary as well as greatly challenging. This also holds true for patients with metastatic urothelial cancer (mUC). Since the first approval of a PD1 targeting immune checkpoint inhibitor (ICI) as second line treatment for patients with mUC, research has been focussed on predictive biomarkers to identify the ~ 20% of the patients who could benefit from this therapy. Although several biomarkers have been studied in patients with UC, only PD‐L1 expression is clinically applicable [1, 2, 3, 4, 5, 6]. However, variable assays, cut‐offs and scoring algorithms, as well as inconsistent findings with respect to the predictive value of PD‐L1 expression hamper patient selection based on PD‐L1 [7, 8, 9, 10, 11, 12, 13]. Other biomarkers currently under investigation include tumour mutational burden (TMB) and chromosomal aneuploidy. Whereas a high tumour mutational burden (TMB) is predictive for a good response to immunotherapy due to the generation of many neo‐epitopes [14, 15, 16], a high level of aneuploidy was found to be associated with tumour evasion and reduced response to immunotherapy [17]. The value of circulating cell‐free DNA (cfDNA) as a biomarker has been shown in UC [18, 19, 20, 21, 22, 23]. More specifically, the fraction of circulating tumour DNA (ctDNA) within the total pool of cfDNA was found to be prognostic. With regard to ICIs, the level of aneuploidy detected in cfDNA through whole genome sequencing (WGS), prior to or during treatment, is also predictive for response in a heterogeneous group of patients with cancer [24]. The modified fast aneuploidy screening test‐sequencing system (mFast‐SeqS) represents an affordable (50 euros per sample at the time of this analysis), low‐resolution assay to detect tumour‐derived aneuploidy in cfDNA of patients with cancer [25]. Here, we aimed to explore whether ctDNA abundance determined using the mFast‐SeqS assay provides a promising strategy for the stratification of patients with mUC prior to ICI treatment. For this purpose, mFast‐SeqS analysis was performed on prospectively collected cfDNA from 74 patients with mUC before start with pembrolizumab treatment.

## 2 Materials and methods

### 2.1 Patients

Patients with advanced or metastatic UC were included in a phase II prospective biomarker discovery study (RESPONDER trial, NCT03263039), which was approved by the local ethics board of the Foundation BEBO (Evaluation of Ethics in Biomedical Research), Assen, The Netherlands. In addition, patients were included in the CPCT‐02 biopsy protocol (Center for Personalized Cancer Treatment (CPCT) consortium, NCT01855477), which was approved by the medical ethics committee of the University Medical Center, Utrecht, the Netherlands. Both trials were designed in accordance with the Declaration of Helsinki. All patients provided written informed consent. Patients were treated with pembrolizumab in a three‐week schedule. Prior to start of therapy and until progressive disease (PD), tumour response was evaluated every 12 weeks using computed tomography (CT). Tumour response was determined according to response evaluation criteria in solid tumours v1.1 (RECIST v.1.1) and defined as stable disease (SD), partial/complete response (PR/CR) or progressive disease (PD). Clinical benefit was defined SD or PR/CR according to RECIST v.1.1 at 6 months and continuation of pembrolizumab beyond 6 months. Patients who did not fulfil these criteria were defined as non‐responders. Overall survival (OS) 6 months after treatment start was also recorded (alive/deceased). Additionally, the following clinical parameters related to response were collected: PD‐L1 status, the presence of visceral metastases, level of lactate dehydrogenase (LDH), platelets, haemoglobin (Hb) level, response to first line chemotherapy (SD, CR/PR, PD), treatment‐free interval of < 3 months after first line chemotherapy, WHO performance score, and albumin level.

### 2.2 Specimen characteristics

Peripheral blood was collected in CELLSEARCH® CellSave preservation tubes (Menarini Silicon Biosystems Inc, Huntington Valley) < 10 days before the start of pembrolizumab treatment. Metastatic tissue biopsies were collected at baseline. Tissue specimens were frozen fresh for WGS and specimens for immunohistochemical staining were formalin‐fixed and paraffin‐embedded (FFPE).

### 2.3 Plasma analysis

Plasma was isolated within 96h after blood collection by performing two sequential centrifugation steps: 10 min at 1711 ***g*** at room temperature (RT), and 10 min at 12 000 ***g*** at 4 °C. After centrifugation, plasma was snap‐frozen and stored at −80 °C until further handling. cfDNA was isolated from 2–4 mL of plasma using the QIAamp circulating nucleic acid kit (Qiagen, Venlo, The Netherlands). To enable aneuploidy analysis, the mFast‐SeqS was used as described by Belic et al. [26]. In short, Line‐1 (L1) amplicon libraries were prepared from 1 ng cfDNA using target‐specific L1 primers and Phusion Hot Start II Polymerase as described before. Resulting PCR products were purified by AMPure Beads (Beckman Coulter) and used for a second PCR, in which sequencing adaptors and sample‐specific indexes were added. Resulting sequencing libraries were quantified using the NEBNext Library Quant Kit for Illumina (New England Biolabs), after which libraries of 20 samples were pooled in equimolar amounts and sequenced on the MiSeq platform (Illumina) generating at least 90,000 single reads of 150 base pairs. The next generation sequencing (NGS) Oncomine™ Colon cfDNA Assay (Thermo Fisher Scientific, Waltham, MA, USA) was used according to the manufacturer’s instructions starting with 10 ng cfDNA to determine variant allele frequencies (VAFs) of known mutations in the cfDNA [27].

### 2.4 Tumour tissue analysis

Tumours (archival tissue or newly obtained tissue specimens whenever feasible) with a PD‐L1 combined positivity score (CPS) ≥ 10 based on the companion diagnostic assay of pembrolizumab (PD‐L1 IHC 22C3 pharmDx, Agilent Technologies, Carpinteria, CA, USA) were defined as PD‐L1 positive. DNA was extracted from freshly obtained metastatic tissue, subsequently sequenced at a median depth of 106x, and aligned to the reference human genome GRCH37 as described previously [28]. Genomic data were requested from the Hartwig Medical Foundation (HMF) and provided under data request number DR‐176.

### 2.5 Data analysis

mFast‐SeqS sequencing results of LINE‐1 elements throughout the genome were mapped on human reference genome hg19 using Burrows–Wheeler alignment (v0.7.17). Per chromosomal arm, a Z‐score (measure for deviation from a reference panel of healthy/diploid subjects) was calculated by subtracting the mean and dividing by the standard deviation of normalized read‐counts for the respective chromosome arm to assess over‐ and under‐representation [26]. Z‐scores per chromosome arm were squared and summed into a genome wide aneuploidy score per patient. Based on the cut‐off as described by Belic et al., a genome wide aneuploidy score was defined as high (≥ 5) or low (< 5) [26]. With respect to the Oncomine™ Colon cfDNA Assay data, we only considered variants with a molecular coverage of at least 100 reads including at least 3 variant reads and a variant allele frequency higher or equal to the limit of detection (LOD) for that specific amplicon as true calls. To evaluate the response (categorized as response vs. non‐response) in association with the aneuploidy score (high vs. low), PD‐L1 status in the tumour (positive vs. negative) tumour mutational burden (TMB; high vs. low) of the tumour, and cfDNA‐based variant detection by Oncomine (yes vs. no), the Chi‐square test was used. In addition, the association between the aneuploidy (high vs. low) and OS at 6 months (deceased vs. alive) was evaluated on the same manner. Kaplan–Meier curves showing the differences in treatment duration (in days) in patients with high vs low aneuploidy scores, and in patients with PD‐L1 positive vs. negative tumours were generated. Variables that reached significance in the univariate logistic regression using the ‘glm’ package were combined into a multivariable model, which was 10‐fold cross‐validated with 3 repeats using the ‘caret’ package [29, 30]. The Spearman correlation coefficient was determined between VAF and aneuploidy score. For tissue, WGS data and copy number alterations (CNAs) were estimated using PURPLE v2.49 [31]. From this data, the relative copy number deviation from the expected normal copy number was calculated per chromosome arm and log_2_ transformed. Tissue‐based CNAs per chromosome arm were correlated with cfDNA‐based mFast‐SeqS results per chromosome arm using Spearman correlation. All analyses were performed using either SPSS (v.25) or the statistical platform R (v3.6.1.) (R Core Team, 2017).

## 3 Results

### 3.1 Baseline patient characteristics

**Fig. 1:**
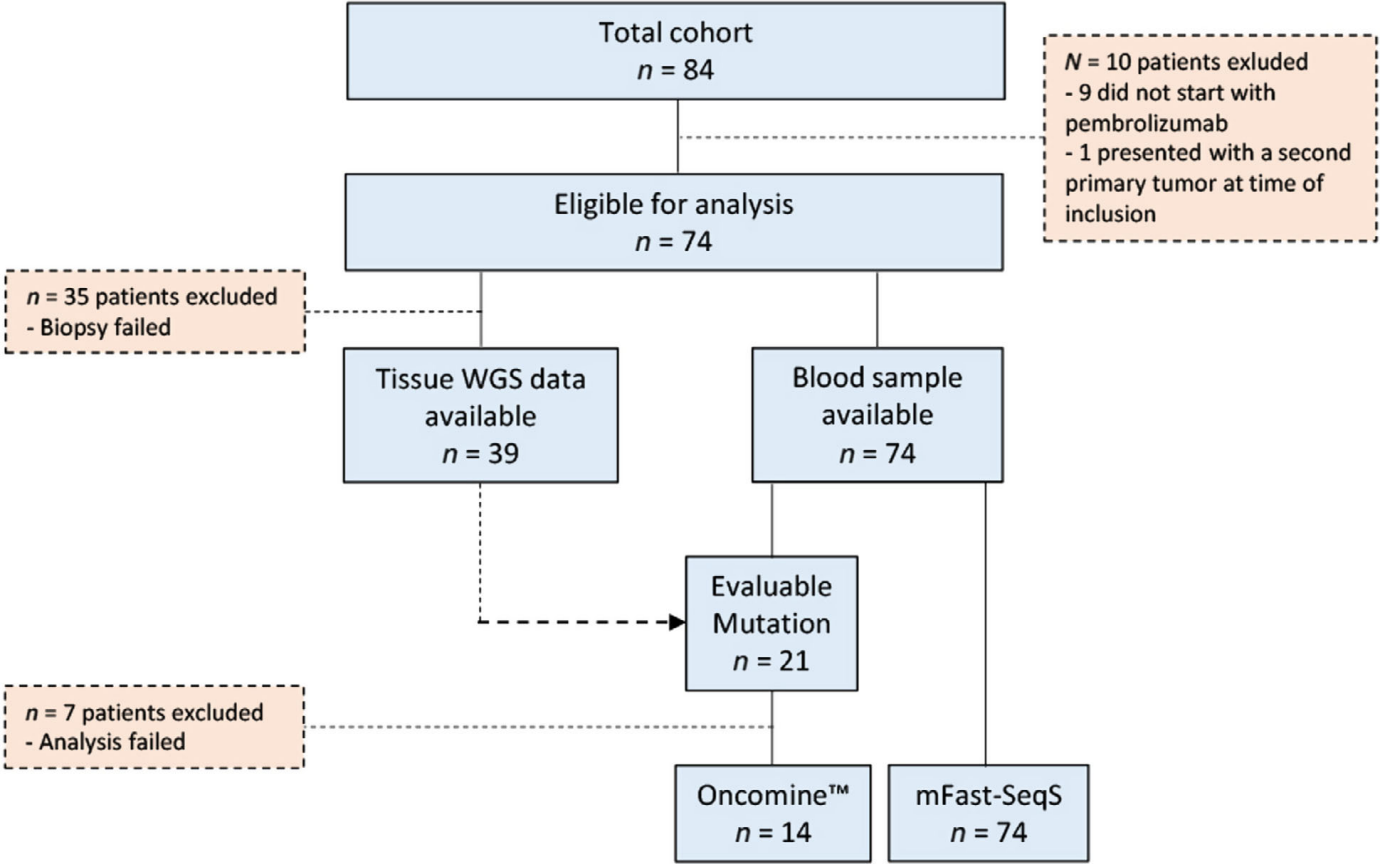
Cohort description. Description of the number of included patients per method of analysis. Per method, the number of excluded patients and the reason of exclusion of samples or patients is mentioned.

All patients who participated in the RESPONDER study (*n* = 84) were initially included. Ten patients were excluded because they did not start pembrolizumab treatment (*n* = 9) or due to the presence of a second primary tumour at baseline (*n* = 1). The final cohort consisted of 74 patients of whom 54 were male and 37 had visceral metastases at inclusion. All patients had WHO performance score of ≤ 1. Patients were eligible to be treated with pembrolizumab in second line (*n* = 64) or in first line in case of cisplatin ineligibility and a CPS ≥ 10 for PD‐L1 in their tumour (*n* = 10). In second line, 23 patients (36%) had a CPS ≥ 10 for PD‐L1. Median number of cycles was 8 (range 1–35). 26 patients were categorized as responders whereas 48 patients were non‐responders. An overview of the cohort is provided in Fig. 1.

### 3.2 mFast‐SeqS detected aneuploidy in the blood is tumour‐derived and reflects the ctDNA level

**Fig. 2:**
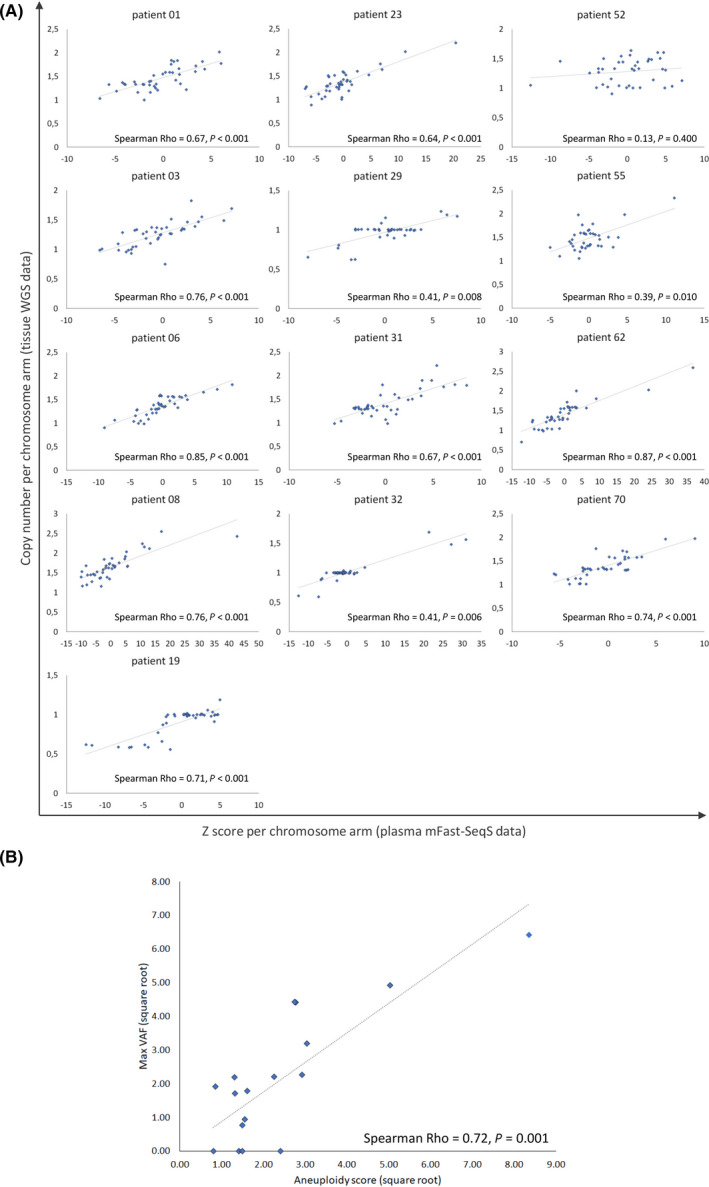
mFast‐SeqS detected aneuploidy in the blood is tumor‐derived and reflects the ctDNA level. (A) 13 patients with aneuploidy score ≥ 5. Spearman correlation coefficient was estimated per chromosomal arm between copy number alterations (CNAs) from WGS data and Z‐score from mFast‐SeqS performed on ctDNA. The relative deviation of CN from the expected normal CN was calculated and log_2_ transformed. (B) 17 patients in which a mutation covered by the Oncomine™ Colon cfDNA Assay was successfully detected. Spearman correlation between the maximal VAF and aneuploidy score per patient. Values were square‐root transformed to reduce skewness in the data distribution.

Tissue‐based WGS data was available for 39 patients out of 74 patients, for the other patients no WGS could be performed due to insufficient biopsy quality (not representative or very few tumour cells). In 13 of these 39 patients we also detected copy number alterations (CNAs) in blood plasma. For these 13 patients, CNAs found in their metastatic tumour biopsies were correlated with the estimated CNAs in cfDNA as determined by mFast‐SeqS. We found a median Spearman Rho of 0.67 (range: 0.13–0.87) between the tissue and blood‐based copy number profiles, and for 12 out of 13 patients this correlation was significant (*P* < 0.01; (Fig. 2A)). Out of the 39 patients from whom tissue‐based WGS data was available, 21 patients had one or more mutations covered by the Oncomine™ Colon cfDNA Assay (Table 1). Subsequent mutation analysis of the cfDNA in these patients was successful in 17 patients carrying in total 22 evaluable mutations. Out of these 22 mutations, 17 (77%) were detected in the cfDNA and the maximal VAF observed in the plasma of these patients correlated well with the aneuploidy score in the same plasma sample (Spearman Rho 0.72 (*P* < 0.001; (Fig. 2B)). Together, these results suggest that the aneuploidy detected in the blood by mFast‐SeqS is derived from the tumour and reflects the level of ctDNA present in the total pool of cfDNA.

**Table 1:** Overview of the observed mutations in tissue‐based WGS data and the corresponding Oncomine NGS results in plasma samples. HGVS.c, Human Genome Variation Society coding mutation nomenclature; VAF, variant allele frequency.

Subject	Gene	HGVS.c	Protein	VAF in tissue	VAF in plasma	Aneuploidy score
2	TP53	c.659A > G	Y220C	52.38	Failed	1.49
3	ERBB2	c.2264T > C	L755S	53.41	19.53	7.7
7	TP53	c.743G > A	R248Q	39.24	Failed	1.06
7	ERBB2	c.929C > T	S310F	27.69	Failed	1.06
10	TP53	c.517G > A	V173M	46.67	Failed	1.22
11	CTNNB1	c.134C > T	S45F	36.36	0	1.99
18	PIK3CA	c.1633G > A	E545K	20.79	Failed	1.24
23	TP53	c.742C > T	R248W	86.84	24.28	25.35
29	FBXW7	c.1514G > A	R505H	30.39	19.61	7.54
32	TP53	c.853G > A	E285K	47.87	41.3	69.89
33	PIK3CA	c.3140A > G	H1047R	30.07	0.87	2.43
33	TP53	c.659A > G	Y220C	30.56	0.9	2.43
41	PIK3CA	c.1633G > A	E545K	31.36	4.83	1.72
41	CTNNB1	c.134C > T	S45F	30.77	3.7	1.72
43	TP53	c.574C > T	Q192Ter	26.60	10.24	9.22
45	FBXW7	c.1513C > G	R505G	38.52	0	0.65
45	PIK3CA	c.1633G > A	E545K	16.54	0	0.65
47	FBXW7	c.1513C > G	R505G	48.98	0	2.23
48	TP53	c.742C > T	R248W	61.33	2.94	1.75
53	CTNNB1	c.110C > G	S37C	38.10	2.69	0.74
53	TP53	c.589G > A	V197M	55.52	3.693	0.74
55	TP53	c.532dup	H178PfsTer3	53.85	0	5.81
60	PIK3CA	c.1624G > C	E542Q	27.73	4.88	5.11
60	PIK3CA	c.1633G > A	E545K	26.92	4.878	5.11
68	TP53	c.517G > A	V173M	47.58	0.6	2.24
70	TP53	c.586C > T	R196Ter	12.64	5.17	8.54
78	PIK3CA	c.1624G > A	E542K	10.99	3.21	2.59

### 3.3 A high blood‐based aneuploidy score at baseline identifies patients unlikely to respond to pembrolizumab

**Fig. 3:**
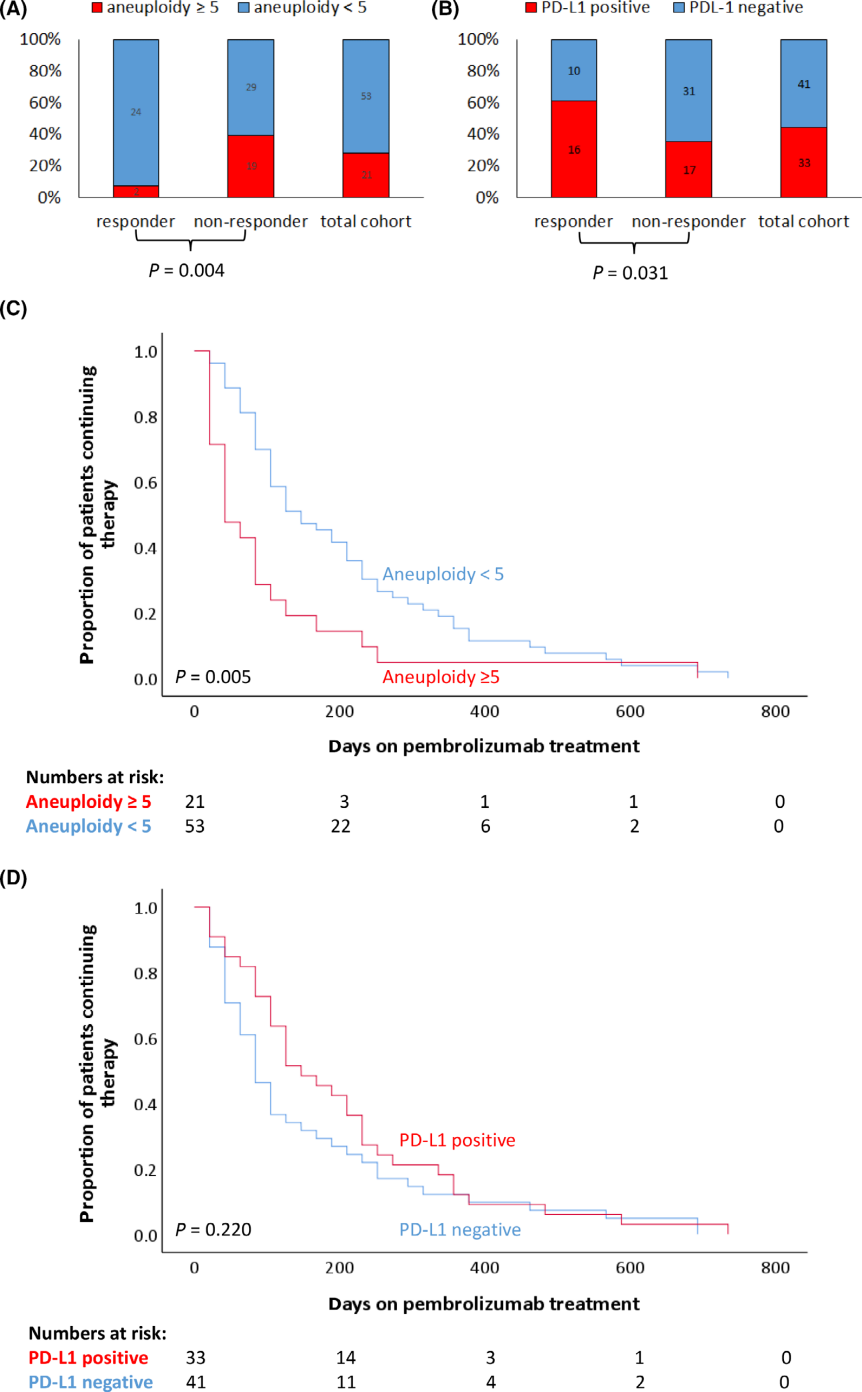
Prediction of response by aneuploidy score and PD‐L1. (A) The proportion of aneuploidy high and low patients was shown for responders, non‐responders, and the total cohort. The Chi‐square test was used to determine statistical significance. (B) The proportion of PD‐L1 positive and negative patients was shown for responders, non‐responders, and the total cohort. The Chi‐square test was used to determine statistical significance. (C) Kaplan–Meier curve is shown for time on pembrolizumab treatment in aneuploidy positive and negative patients. The obtained curves were compared using the log‐rank test. (D) Kaplan–Meier curve is shown for time on pembrolizumab treatment in PD‐L1 positive and negative patients. The obtained curves were compared using the log‐rank test.

As summarized in Figure 3A, mFast‐SeqS analysis resulted in a high aneuploidy score (≥ 5) for 21 patients (28.4%). Clinical benefit was only observed in 9.5% of these patients, whereas 45.3% of patients with a low (< 5) aneuploidy score at baseline showed clinical benefit. This results in a positive predictive value of 0.9 for non‐response with a specificity and sensitivity of 92% and 40% respectively. In comparison, the presence of a positive PD‐L1 CPS in 33 patients (44.6%) resulted in a positive predictive value of 0.76 for response with a sensitivity and specificity of 63% and 66%, respectively (Fig. 3B). Kaplan–Meier analyses showed that time on treatment was significantly shorter in patients with a high aneuploidy score compared to patients with a low aneuploidy score (median time on treatment 42 days (range 21‐693) versus 147 days (range 21‐735); log‐rank test *P* = 0.005) (Fig. 3C). In contrast, time on treatment was not significantly different between patients with a PD‐L1 positive and negative tumour (median time on treatment 147 days (range 21‐735) versus 84 days (range 21‐693); log‐rank test *P* = 0.220) (Fig. 3D). Univariate logistic regression showed that high aneuploidy score, a treatment‐free interval < 3 months and a low/negative PD‐L1 CPS were associated with non‐response on pembrolizumab treatment (*P* < 0.05) (Table 2). When these variables were combined in a multivariate analysis, a treatment‐free interval < 3 months and the aneuploidy score remained significant (Odds Ratio 0.11; *P* = 0.007), indicating that the aneuploidy score represents an independent variable associated with outcome on pembrolizumab (Table 2). For 66 patients, OS at 6 months was known (deceased/alive) and for these patients we performed uni‐ and multivariate logistic regression using this outcome parameter as well (Table 3). Similar to our results for response to pembrolizumab, both a treatment‐free interval < 3 months and the aneuploidy score were significantly associated with OS in multivariate analysis as well.

**Table 2:** Logistic regression model for prediction of clinical benefit. TFI, treatment free interval following first line chemotherapy (at least 4 cycles of cisplatin/gemcitabine or carboplatin/gemcitabine).

	Univariate analysis			Multivariate analysis		
	OR	95% CI	*P* value	OR	95% CI	*P* value
Clinical variables						
Gender	0.36	0.09–1.15	0.105			
Visceral metastases present	0.62	0.23–1.62	0.332			
CPS PD‐L1 ≥ 10	2.92	1.10–8.05	0.034	2.80	0.93–8.92	0.072
Albumin (mg·L^−1^)	1.12	1.00–1.27	0.065			
LDH (U·L^−1^)	0.99	0.98–1.00	0.183			
Platelets (×10^9·L^−1^)	1.00	0.99–1.00	0.115			
Hb (mmol·L^−1^)	1.15	0.74–1.80	0.528			
TFI < 3 months	0.17	0.025–0.66	0.025	0.12	0.02–0.52	0.011
Response to first line chemotherapy	1.49	0.74–3.22	0.275			
mFast‐SeqS variables						
cfDNA concentration (ng·mL^−1^)	0.97	0.93–1.00	0.143			
Aneuploidy score ≥ 5	0.13	0.02–0.50	0.009	0.11	0.02–0.46	0.007
Aneuploidy score continuous	0.89	0.77–0.98	0.066			

**Table 3:** Logistic regression model for prediction of overall survival (OS) at 6 months (deceased vs alive). TFI, treatment free interval following first line chemotherapy (at least 4 cycles of cisplatin/gemcitabine or carboplatin/gemcitabine).

	Univariate analysis			Multivariate analysis		
	OR	95% CI	*P* value	OR	95% CI	*P* value
Clinical variables						
Gender	0.36	0.12–1.07	0.071
Visceral metastases present	0.29	0.10–0.78	0.017	1.43	0.30–7.65	0.660
CPS PD‐L1 ≥ 10	1.93	0.73–5.31	0.193			
Albumin (mg·L^−1^)	1.14	1.03–1.29	0.023	1.10	0.93–1.34	0.300
LDH (U·L^−1^)	1.00	0.99–1.00	0.256			
Platelets (×10^9·L^−1^)	1.00	0.99–1.00	0.382			
Hb (mmol·L^−1^)	1.44	0.91–2.34	0.129			
TFI < 3 months	0.19	0.05–0.66	0.013	0.11	0.01–0.65	0.027
Response to first line chemotherapy	1.90	0.95–4.01	0.076			
mFast‐SeqS variables						
cfDNA concentration (ng·mL^−1^)	0.94	0.89–0.98	0.016	0.92	0.84–0.99	0.056
Aneuploidy score ≥ 5	0.13	0.03–0.41	0.001	0.04	0.003–0.26	0.003
Aneuploidy score continuous	0.79	0.66–0.91	0.005			

Since tissue‐based WGS data and cfDNA‐based mutation data was only available for part of the patients we could not include these variables in our logistic regression models. However, we have compared the proportion of patients with a high (≥ 10) and low TMB burden and the proportion of patients in which a known variant was detected in the blood between responders and non‐responders (Fig. S1). These data support the already established link between TMB and response to ICI [14, 15, 16], but do not show a significant difference in response rate between patients with and without detectable ctDNA by mutation analysis. However, it should be noted that patient numbers for variant analysis by Oncomine were very low.

## 4 Discussion

We show that a high mFast‐SeqS aneuploidy score in baseline plasma samples is indicative of a high ctDNA fraction and represents an independent marker associated with lack of clinical benefit in patients with mUC treated with pembrolizumab. To date, no robust clinical biomarker to stratify for response to pembrolizumab in patients with mUC is available. Although PD‐L1 expression is being used as a biomarker to select first‐line cisplatin‐unfit patients for treatment with a PD(L)1 targeting ICI, its value remains questionable [32, 33]. The prognostic or predictive value of mutation‐based ctDNA detection in UC patients in both the adjuvant and metastatic setting has been shown before [18, 19, 20, 21, 23, 34]. Vandekerkhove et al., previously showed using a mutation‐based assay with a sensitivity of 1% that having a VAF > 5% was prognostic in mUC patients, suggesting that detecting very low levels of ctDNA may not be necessary to achieve clinical relevance [35]. In addition, the use of somatic mutations to estimate the amount of ctDNA requires prior knowledge on the mutation status of a patient’s tumour to discriminate between false negative results and true absence of ctDNA. Finally, the genetic heterogeneity of mUC necessitates large sequencing panels, rendering this approach expensive. To ultimately enable successful clinical implementation of a biomarker, a straightforward, affordable and clinically applicable method is crucial as well. Chromosomal instability detected in liquid biopsies has been described as a possible biomarker in several types of cancer [24, 36]. In line with this, our data show that a simple blood draw in combination with the aneuploidy‐based mFast‐SeqS method could support physicians in their treatment decisions regarding patients with mUC. As with most methods, a possible weakness of the mFast‐SeqS could be the choice of an arbitrary threshold above which samples are defined aneuploidy positive. Our used cut‐off was previously found to equal the presence of 5‐10% ctDNA, which was described as prognostic in mUC [23, 26, 35]. Multivariate logistic regression analysis confirmed that, next to a treatment‐free interval of less than 3 months, the mFast‐SeqS‐based aneuploidy score represents an independent variable associated with both response to pembrolizumab and overall survival at 6 months in patients with mUC. mFast‐SeqS‐based low‐resolution copy number profiles on cfDNA resemble those obtained by genome‐wide sequencing in metastatic tissue, are concordant with genome‐wide sequencing approaches on plasma [26] and can be obtained at a fraction of the costs (in our hands less than 50 euros per sample) from only 0.5‐1 ng of cfDNA, rendering this method feasible for virtually all patients from whom 1 mL of plasma is available. The question remains how to implement this test into daily clinical practice. As said, the prognostic value of ctDNA abundance in mUC was shown before [20, 35, 37], indicating the need for new clinical trials specifically for these patients with a poor prognosis to evaluate treatment options, which are currently not available in the second line of treatment. Based on our results, the mFast‐SeqS assay could provide a promising tool to enable patient selection for these future trials. Ultimately, results from these trials are expected to improve the cost‐effectiveness of treatment by reducing the time to the administration of an effective treatment while at the same time sparing patients unnecessary toxicity.

## 5 Conclusion

In conclusion, we confirmed our hypothesis that the low‐cost and minimally invasive mFast‐SeqS approach on liquid biopsies can be used to predict lack of clinical benefit prior to initiation of pembrolizumab treatment in patients with mUC. After validation in an independent prospective cohort of patients with mUC, implementing this test at baseline may therefore enable early stratification of patients to increase the current treatment success rate and reduce unnecessary toxicity and costs.

## Conflict of interest

The authors have no conflicts of interest to declare.

### Peer Review

The peer review history for this article is available at https://publons.com/publon/10.1002/1878‐0261.13196.

## Author contributions

Conceptualization was done by DR, PAJM, MR, RdW, SMW, and MLHMW, MJBA, JV, and AAMvdV are main clinical contributors. TD and VdW performed the laboratory analyses. SMW, TD, and JAN‐G performed the bioinformatics analyses. DR, PAJM, and MR. managed clinical data assessment. PAJM, DR and SMW wrote the manuscript, which all authors reviewed. MPL is a member of the CPCT‐02 study team and/or CPCT board.

## Supporting information


**Fig. S1.** Positivity rates for tissue‐based TMB, cfDNA‐based mFast‐SeqS aneuploidy score and cfDNA‐based mutation detection in responding and non‐responding patients.

## Data Availability

The WGS data used in this study was made available by the Hartwig Medical Foundation (Dutch nonprofit biobank organization) after signing a license agreement stating data cannot be made publicly available via third party organizations. Therefore, the data are available under restricted access and can be requested upon by contacting the Hartwig Medical Foundation (https://www.hartwigmedicalfoundation.nl/applying‐for‐data/) under the accession code DR‐176.
