# Supplementary material for: Genome‐wide aneuploidy detected by mFast‐SeqS in circulating cell‐free DNA is associated with poor response to pembrolizumab in patients with advanced urothelial cancer
Source: Mol Oncol. 2022 Mar 17;16(10):2086–97. doi: 10.1002/1878-0261.13196 (PMC9120908; doi:10.1002/1878-0261.13196)
Supplement: Supplementary file 1 — Fig. S1. Positivity rates for tissue‐based TMB, cfDNA‐based mFast‐SeqS aneuploidy score and cfDNA‐based mutation detection in responding and non‐responding patients. [file MOL2-16-2086-s001.pdf]

Supplementary Figure 1

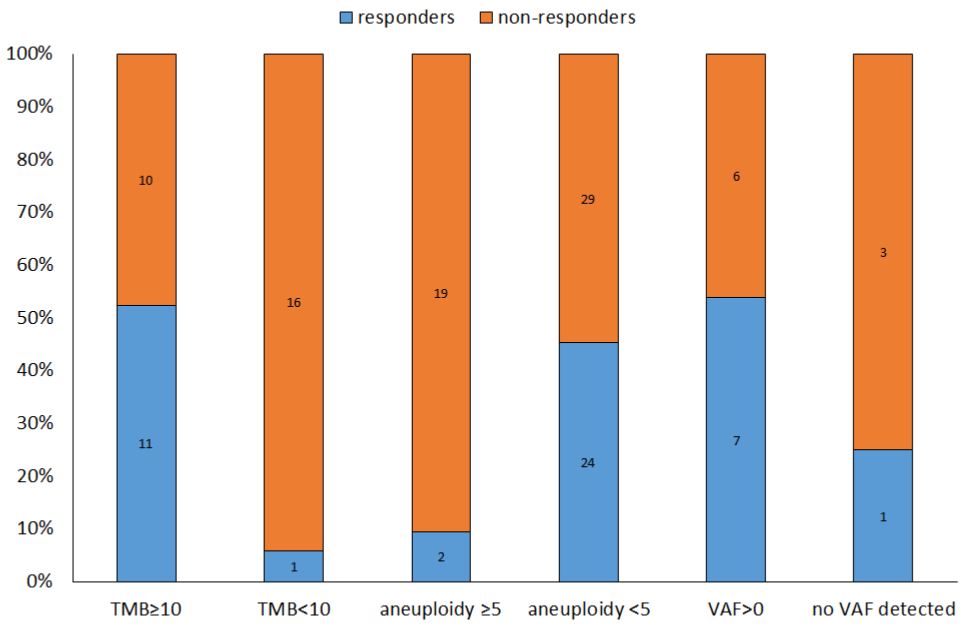

	responders		non-responders		total cohort		evaluated	p value
	%	number	%	number	%	number		
TMB>10	52.38	11	47.62	10	55.26	21	n = 38	0.002
TMB<10	5.88	1	94.12	16	44.74	17		
aneuploidy ≥5	9.52	2	90.48	19	28.38	21	n = 74	0.004
aneuploidy <5	45.28	24	54.72	29	71.62	53		
VAF>0	53.85	7	46.15	6	76.47	13	n = 17	0.312
no VAF detected	25.00	1	75.00	3	23.53	4		

Supplementary Figure 1 – Positivity rates for tissue-based TMB, cfDNA-based mFast-SeqS aneuploidy score, and cfDNA-based mutation detection in responding and non-responding patients

A. The proportion of responders and non-responders is shown for patients with a high (≥10) and low (<10) tumor mutational burden (TMB) in their tissue-based WGS data, patients with a high (≥5) and low (<5) mFast-SeqS-based aneuploidy score in their cfDNA, and patients with and without a detectable variant allele frequency (VAF) in their cfDNA for a mutation known to be present in the tumor based on tissue-based WGS data.

B. Comparison between the number of marker-positive and -negative patients between responders and non-responders using the Chi-square test.
